# Upregulation of Ets1 expression by NFATc2 and NFKB1/RELA promotes breast cancer cell invasiveness

**DOI:** 10.1038/s41389-018-0101-3

**Published:** 2018-11-23

**Authors:** Gi-Cheon Kim, Ho-Keun Kwon, Choong-Gu Lee, Ravi Verma, Dipayan Rudra, Taemook Kim, Keunsoo Kang, Jong Hee Nam, Young Kim, Sin-Hyeog Im

**Affiliations:** 1 Division of Integrative Biosciences and Biotechnology (IBB), Department of Life Sciences, Pohang University of Science and Technology, Pohang, 37673 Republic of Korea; 2 Department of Microbiology and Immunobiology, Harvard Medical School, Boston, MA 02115 USA; 3 Department of Biological Sciences, Korea Advanced Institute of Science and Technology, Daejeon, 34141 Republic of Korea; 4 Department of Microbiology, College of Natural Sciences, Dankook University, Cheonan, 31116 Republic of Korea; 5 Chonnam National University Medical School, Gwangju, 501-749 Korea; 6 Department of Oral Pathology, School of Dentistry, Chonnam National University, Gwangju, 501-749 Korea

## Abstract

Breast cancer is highly aggressive and is the leading cause of cancer-related mortality in women in developed countries. The ETS proto-oncogene 1 (Ets1) has versatile roles during the cellular processes of cancer development. It is often highly expressed in breast cancers and mediates migration and invasion of human breast cancer cells. However, underlying mechanisms of *Ets1* gene expression is still ambiguous. Here, we identified a core-regulatory element (CRE) located in the Ets1 promoter region (−540/−80 bp from TSS) that contains elements responsible for associating with NFATs and NF-κBs. Compared with the less metastatic breast cancer cells, metastatic breast cancer cells (MDA-MB-231) show open chromatin configurations in the CRE, which facilitates direct binding of NFATc2 and/or NFKB1/RELA complex to trans-activate *Ets1* transcription. Moreover, enhanced level of *Nfatc2* and *Nfkb1* positively correlated with Ets1 expression in the human breast cancer specimens. Deletion of the CRE region by CRISPR/Cas9 system resulted in significant reduction in Ets1 expression, which led to alterations of Ets1-mediated transcription programs including tumor invasiveness-related genes. Proper regulation of *Ets1* gene expression by targeting the NFATc2 and NFKB1/RELA interaction could be a potential therapeutic target for Ets1-mediated metastatic breast cancer.

## Introduction

Cancer cells have unique programs to potentiate tumorigenesis at the transcriptional, post-transcriptional and post-translational steps^1^. The ETS proto-oncogene 1 (Ets1) is known as an oncogenic transcription factor. Ets1 contributes to the development and progression of diverse tumors such as epithelial tumor, sarcomas, and astrocytomas^2–4^ by directly regulating the expression of extracellular matrix remodeling factors such as MMP-1, MMP-3 and MMP-9, and uPA (urokinase-type plasminogen activator)^5–8^. Ets1 also promotes the angiogenic process of tumor cells by enhancing the expression of vascular endothelial growth factor (VEGF) receptor, Neuropilin-1 (Nrp1), and angiopoietin-2 (Ang2)^9–12^. Ets1 also regulates epithelial–mesenchymal transition (EMT) in epithelial and carcinoma cells^13,14^. Moreover, high level of Ets1 expression was closely linked with strong metastatic potential and poor clinical prognosis in various types of cancers^15–17^. Accordingly, Ets1 could be a conceivable therapeutic target especially in the triple-negative/basal-like breast cancers (TN/BLBC) that show Ets1^high^ expression profile compared with non-TNBC cells^18^.

Interestingly, however, underlying mechanisms of transcriptional regulation of *Ets1* gene expression is poorly characterized in cancer cells. Previous studies were mainly focused on understanding how Ets1 expression is regulated by factors within tumor microenvironment such as hepatocyte growth factor (HGF), basic fibroblast growth factor (bFGF), vascular endothelial growth factor (VEGF), Platelet-derived growth factor-BB (PDGF-BB), and transforming growth factor beta (TGFβ)^19–22^. These extrinsic factors enhance *Ets1* transcription through subsequent activation of downstream signaling pathways including MEK/ERK1/2, PI3K (phosphoinositol-3-kinase)/AKT, protein kinase C (PKC), and calcium signaling^19–23^. Under such conditions, several transcription factors (such as AP-1, Ets1, and hypoxia-mediated HIF1α [HIF1α]) are known to directly upregulate *Ets1* transcription in cancer cells^24–26^. However, it is still unclear which types of transcriptional factors and *cis*-acting regulatory elements cooperatively regulate transcriptional activity of *Ets1* gene expression, especially in breast cancer cells.

In this study, we investigated the transcriptional and epigenetic regulation of *Ets1* gene expression in metastatic breast cancer cells. We identified a core-regulatory element (CRE) on the *Ets1* promoter and elucidated its functional importance in tumor invasiveness. Compared with less metastatic cells (MCF-7), metastatic breast cancer cells (MDA-MB-231) have relatively open chromatin structure on the CRE, which facilitates direct binding of NFATc2 and NFKB1/RELA to enhance Ets1 expression and invasiveness of metastatic breast cancers, accordingly.

## Results

### Ets1 expression is regulated at the transcriptional level in breast cancer cells

**Fig. 1:**
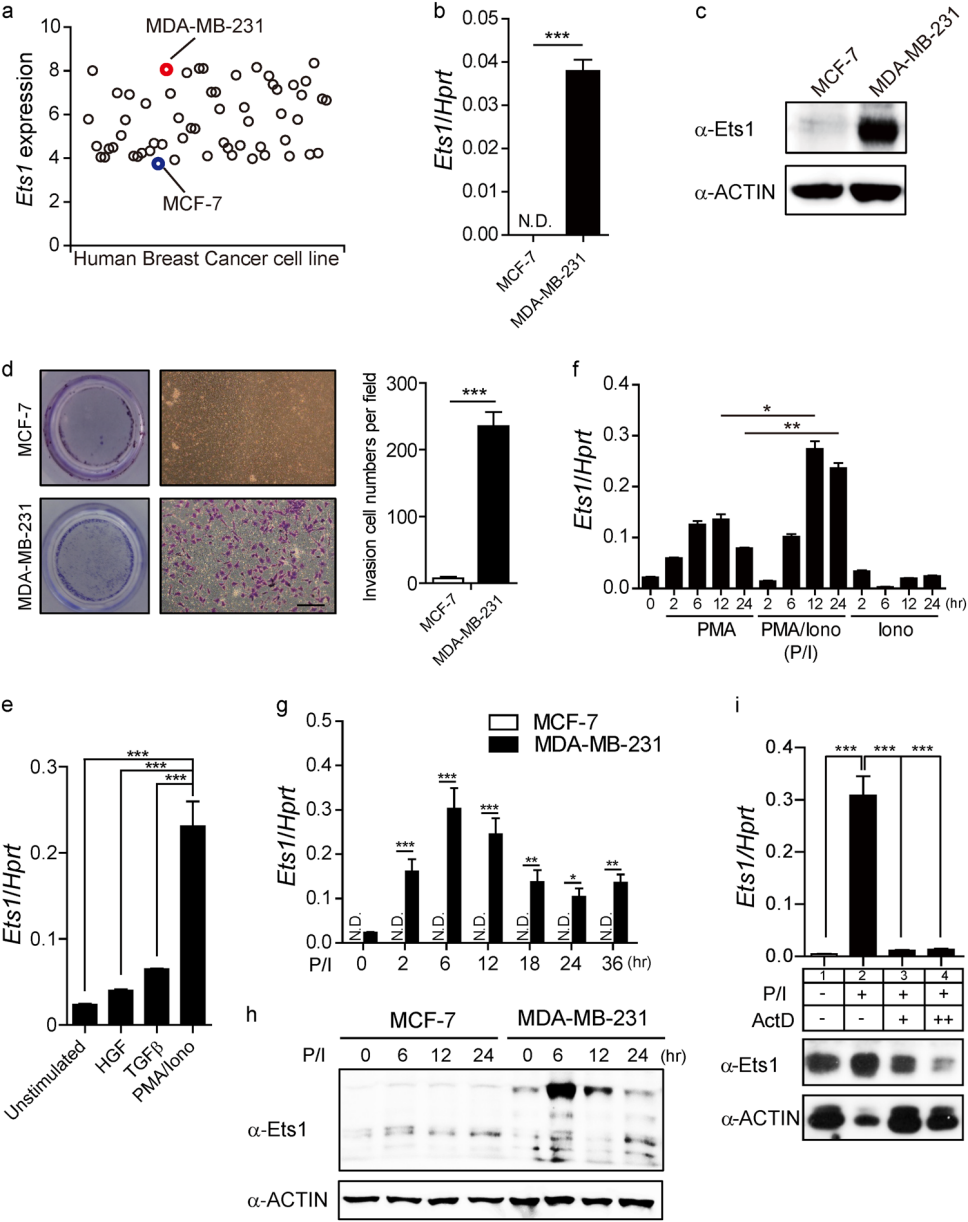
Comparative analyses of Ets1 expression between metastatic MDA-MB-231 and less metastatic MCF-7 breast cancer cells. **a** Analysis of *Ets1* expression profile in 59 breast cancer cell lines by Cancer Cell Lines Encyclopedia (CCLE). **b**, **c** Analyses of Ets1 transcripts and protein levels by qRT-PCR (**b**) and Immuno-blot (**c**) in unstimulated condition. **d** Cells were stained with crystal violet and representative images were obtained from in vitro invasion assay using 10% FBS as chemoattractant. Scale bar: 100 m. **e** Metastatic MDA-MB-231 cells were treated with indicated stimuli for 6 h and relative levels of *Ets1* transcripts normalized against *Hprt* are shown. **f** Effect of PMA (**p**), Ionomycin (**i**) and their combination (**p**/**i**) on *Ets1* transcripts levels determined by qRT-PCR. **g**, **h** Comparative analysis of Ets1 transcripts and protein level between the cells in response to PMA/Ionomycin (**p**/**i**) stimulation. Data are presented as mean ± SD. Two-way ANOVA with Bonferroni post-tests showed a significant difference of Ets1 expression. **i** Effect of actinomycin D (Act D) treatment in MDA-MB-231 cells on *Ets1* transcripts and protein levels determined by qRT-PCR and Immuno-blot, respectively. Values in **b**, **d**, **f**, **e**, and **i** are means ± SD. One-way ANOVA with Bonferroni correction indicated a significant difference. **b**–**i** Data shown are representative of more than three independent experiments with similar results. N.D. not detected. *p < 0.05, **p < 0.01, and ***p < 0.001

To understand the transcriptional regulation mechanisms of *Ets1* expression in breast cancer cells, we first analyzed *Ets1* transcript level among various breast cancer cell lines. Based on *Ets1* level, cancer cells were divided into two categories: Ets1^high^ and Ets1^low^ cell lines (Fig. 1a, Supplementary Figures S1a, b). We chose three representative cell lines, MCF-7 (Ets1^low^), MDA-MB-468(Ets1^low^), and MDA-MB-231 (Ets1^high^), and confirmed the expression status of Ets1 by qRT-PCR and Immuno-blot (Fig. 1b, c) (Supplementary Figures S1a, b). Since Ets1 expression is correlated with invasiveness of tumor cells^27^, we compared the invasive properties of MCF-7 and MDA-MB-231 by invasion assay. Indeed, MDA-MB-231 (Ets1^high^) cells were more invasive than MCF-7 (Ets1^low^) cells (Fig. 1d). To confirm this observation is Ets1-dependent, we compared non-metastatic MDA-MB-468 cells with MDA-MB-231 cells, which share similar hormonal status. Similar to the MCF-7 cells, MDA-MB-468 cells showed reduced Ets1 expression with less invasive properties than MDA-MB-231 cells (Supplementary Figures S1a, b).

Next we tested which types of stimuli trigger *Ets1* expression in MDA-MB-231 cells. To mimic tumor microenvironment (TME), MDA-MB-231 cells were stimulated with various tumorigenic factors such as TGFβ, HGF, and PMA and Ionomycin^28–30^. Co-treatment with PMA and Ionomycin (P/I) induced the highest level of *Ets1*, indicating synergy between PKC and calcium (Ca^2+^) pathways (Fig. 1e, f). Compared with MCF-7 cells, MDA-MB-231 cells showed significantly enhanced Ets1 expression in mRNA and protein levels upon PMA/Ionomycin stimulation (Fig. 1g, h). Treatment of the transcription inhibitor actinomycin D abolished PMA/Ionomycin induced Ets1 expression, indicating that Ets1 expression is regulated at the transcriptional level in the metastatic MDA-MB-231 breast cancer cells (Fig. 1i).

### Identification of core-regulatory element (CRE) and CRE-binding transcription factors involved in Ets1 expression

**Fig. 2:**
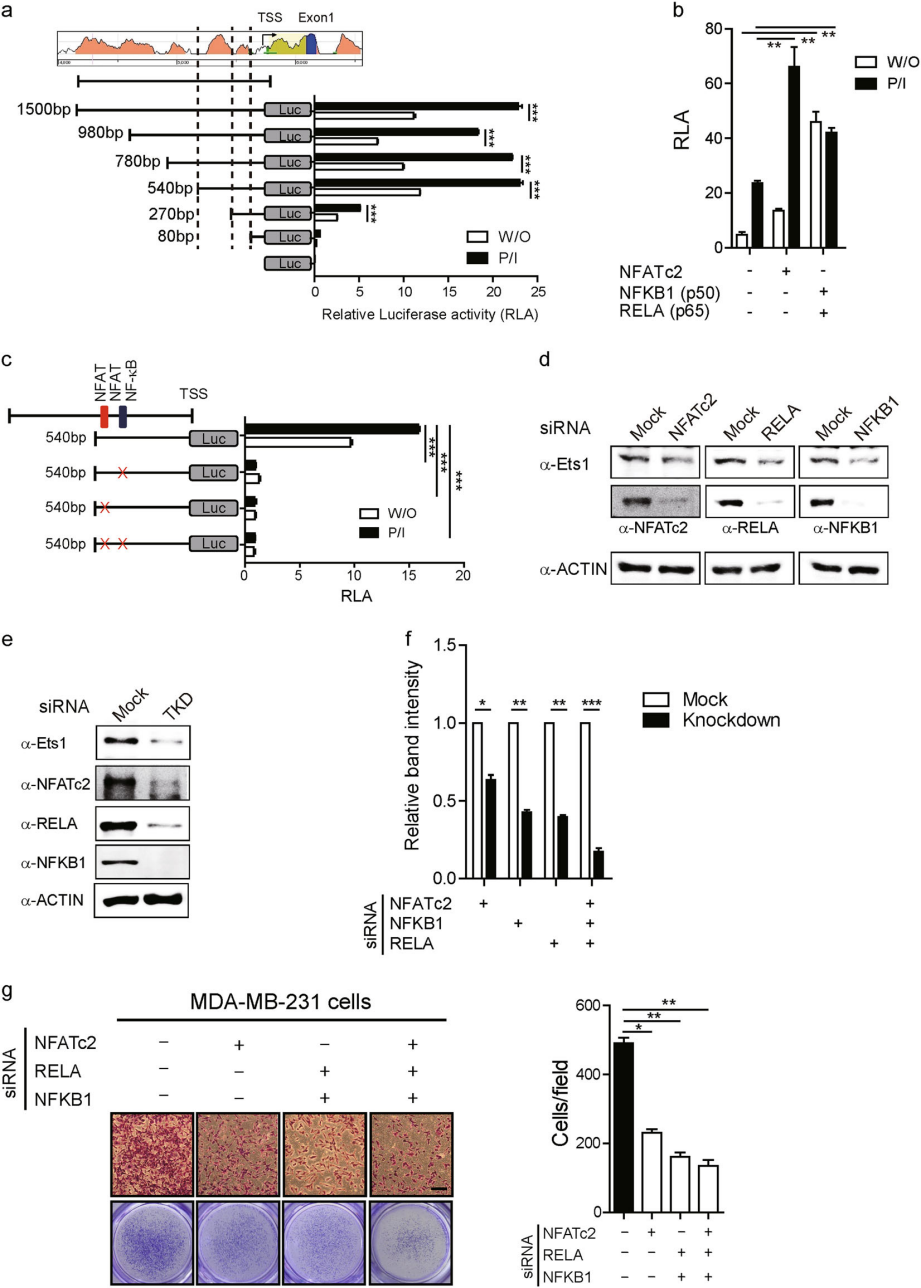
Identification of core-regulatory element (CRE) and CRE-binding transcription factors involved in Ets1 expression. **a** Schematic diagram of the genomic position and size of the deletion constructs of promoter region of *Ets1* gene are shown. **b** MDA-MB-231 cells were transfected with Ets1 promoter-Luc reporter vector (540 bp) and together with indicated combinations (minus, plus) of expression vectors. Relative luciferase activity was measured. **c** MDA-MB-231 cells were transfected with Ets1 promoter-Luc (540 bp) vector or vectors mutated in the NFAT or NFAT/NFKB binding sites (multiply: mutation site). Relative luciferase activity in response to PMA/Ionomycin stimulation was measured. **b**, **c** Relative luciferase activities relative to the expression of Renilla luciferase plasmid (hRluc) are calculated as fold difference relative to the control value. **d**, **e** Effects of knockdown of indicated transcription factors on the expression of Ets1. MDA-MB-231 cells were transfected with mock siRNAs or siRNAs for indicated transcription factors (NFATc2, NFKB1 and RELA) (**d**) or triple combination of them (TKD) (**d**, **e**). Knockdown efficiency on the level of transcription factors by individual siRNAs or TKD siRNAs was confirmed by Immuno-blot (**f**) and band intensity of Immuno-blot was quantified by ImageJ software. **g** Knockdown of individual siRNAs or TKD siRNAs on invasive properties of MDA-MB-231 cells by invasion assay. Scale bar: 100 m. Data are presented as mean ± SD. One-way ANOVA with Bonferroni correction indicated a significant difference of invaded cells. **a**–**c**, **f** Data are presented as mean ± SD. Two-way ANOVA with Bonferroni post-tests showed a significant difference. **a**–**g** Data shown are representative of more than three independent experiments with similar results. *p < 0.05, **p < 0.01, and ***p < 0.001

To identify the core-regulatory element (CRE), we serially deleted the Ets1 promoter region and tested its effect on trans-activation of *Ets1* expression measured by reporter activity. As shown in Fig. 2a, deletion of −540 to −80 bps from transcription start site (TSS) significantly reduced the luciferase activity, indicating the pivotal role of this locus for the trans-activation of Ets1 expression. Using an ECR browser^31^, we identified NFATs and NF-κB transcription factors could potentially bind to this locus to activate Ets1 expression (Supplementary Figures S2a, b). Indeed, among the diverse transcription factors tested, co-transfection of NFATc2 or NFKB1/RELA resulted in highest Ets1 promoter activity (Fig. 2b, Supplementary Figure S2c). In contrast, mutations of NFAT or NF-κB binding sequences completely abrogated Ets1 promoter activity (Fig. 2c). We also confirmed that knockdown (KD) of these factors (*Nfatc2*, *Nfkb1*, and *Rela*) significantly reduced Ets1 expression (Fig. 2d–f). Moreover, triple knockdown (TKD) of these factors most significantly reduced Ets1 expression (Fig. 2e, f) and decreased the invasive properties of MDA-MB-231 cells (Fig. 2g). These results indicate NFATc2, NFKB1 and RELA cooperatively enhance Ets1 expression through CRE (−80 to −520bps from TSS) region in the metastatic MDA-MB-231 breast cancer cells.

### NFATc2 and NFKB1/RELA regulate Ets1 expression in metastatic breast cancer cells

**Fig. 3:**
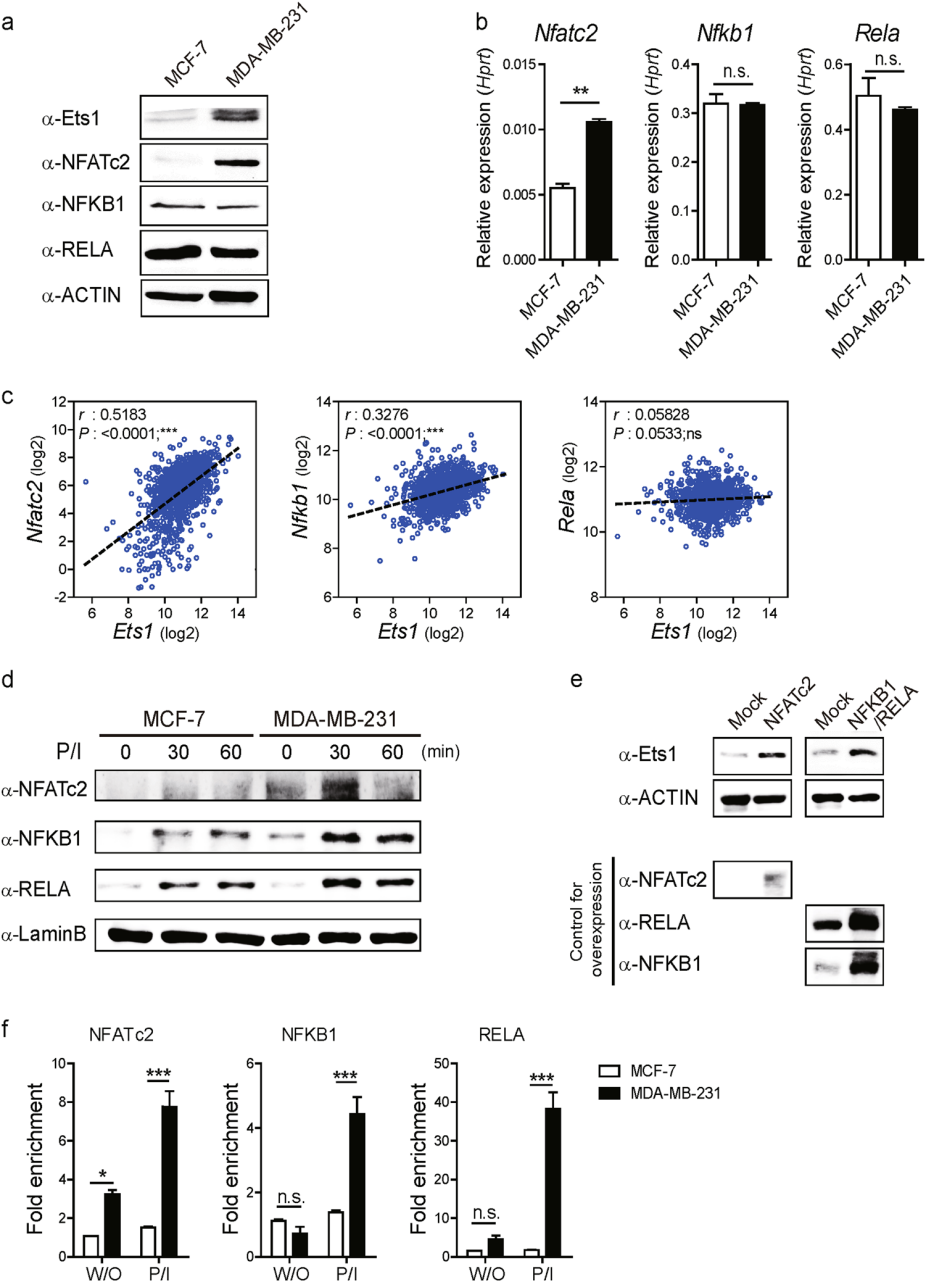
NFATc2 and NFKB1/RELA regulate Ets1 expression in breast cancer cells. **a**, **b** Comparison of NFATc2, NFKB1, and RELA protein and transcript levels in whole cell lysates of MCF-7 and MDA-MB-231 cells determined by **a** Immuno-blot and **b** qRT-PCR normalized against *Hprt*. Data are presented as mean ± SD. One-way ANOVA with Bonferroni correction indicated a significant difference of expression. **c** Scatterplots and Spearman’s rank correlation from TCGA database. Correlation between mRNA expression of *Ets1* with *Nfat*c2, *Nfkb1*, and *Rela*. Each symbol represents an individual human specimen. **a**, **d** Representative Immuno-blot for the total and nuclear (NE) protein levels in unstimulated conditions or cells stimulated for indicated time points. ACTIN and LaminB1 served as loading control for total and NE, respectively. **e** Effect of overexpression of transcription factors (NFATc2 and NFKB1/RELA) on Ets1 expression in MCF-7 cells determined by Immuno-blot. **f** Relative enrichment of transcription factors to the CRE region determined by ChIP analysis with specific antibodies for NFATc2, NFKB1, and RELA. Results are presented relative to input DNA. Data are presented as mean ± SD. Two-way ANOVA with Bonferroni post-tests showed a significant difference of binding efficiency of NFATc2, NFKB1, and RELA between MCF-7 and MDA-MB-231 cells. **a**, **b**, **d**, **e**, **f** Data shown are representative of more than three independent experiments with similar results. *p < 0.05, **p < 0.01, and ***p < 0.001 (unpaired *t*-test)

To gain further mechanistic insight on the role of NFATc2 and NFKB1/RELA on Ets1 expression in MCF-7 (Ets1^low^) and MDA-MB-231 (Ets1^high^) cells, we next compared the expression status of NFATc2 and NFKB1/RELA in these cell lines. MDB-MB-231 cells expressed much higher levels of NFATc2 in mRNA and protein (total and nuclear) compared with MCF-7 cells (Fig. 3a, b, d). On the other hand, no significant differences were observed in NFKB1 and RELA levels between these cells (Fig. 3a, b). Similar to MCF-7 cells, non-metastatic triple-negative MDA-MB-468 cells showed reduced expression of NFATc2 levels and invasion related genes including *Eng* and *Mmp14* compared to MDA-MB-231 cells (Supplementary Figures S1c, d).

Next, we tested whether differential expression of NFATc2 or NF-κBs has any correlation with Ets1 expression in human breast cancer specimens by analyzing The Cancer Genome Atlas (TCGA) database that provide unbiased large-scale transcriptome data for various human cancer^32^. Indeed, expression level of *Nfatc2* (Spearman *r* = 0.5183, ***p < 0.0001) and *Nfkb1* (Spearman *r* = 0.3276, ***p < 0.0001) but not *Rela* showed correlative expressions with *Ets1* levels in human specimens (Fig. 3c). We also compared nuclear levels of NFATc2, NFKB1, and RELA levels between the cells. Compared with MCF-7 cells (Ets1^low^), MDA-MB-231 (Ets1^high^) cells showed significantly higher levels of NFATc2, NFKB1, and RELA in the nucleus upon PMA/Ionomycin stimulation (Fig. 3d). Forced expression of NFATc2, NFKB1/RELA in MCF-7 cells significantly enhanced Ets1 expression and invasiveness (Fig. 3e, Supplementary Figure S3a). Overexpression of Ets1 also enhanced the invasive capacity of MCF-7 cells (Supplementary Figure S3b). Next, we performed ChIP assay to measure the binding of these factors to CRE region between the cells. Compared with MCF-7 cells (Ets1^low^), MDA-MB-231 (Ets1^high^) cells showed higher binding of NFATc2 as well as NFKB1/RELA to the CRE region (Fig. 3f). In addition, overexpression of NFATc2 and NFKB1/RELA increased the binding of these factors to the CRE region in MCF-7 cells (Supplementary Figure 3c). These results suggest that cellular availability as well as relative enrichment of NFATc2 and NFKB1/RELA to the CRE region mediate differential Ets1 expression between MDA-MB-231 (Ets1^high^) and MCF-7 cells (Ets1^low^).

### Permissive chromatin architecture at the promoter is required for Ets1 expression

**Fig. 4:**
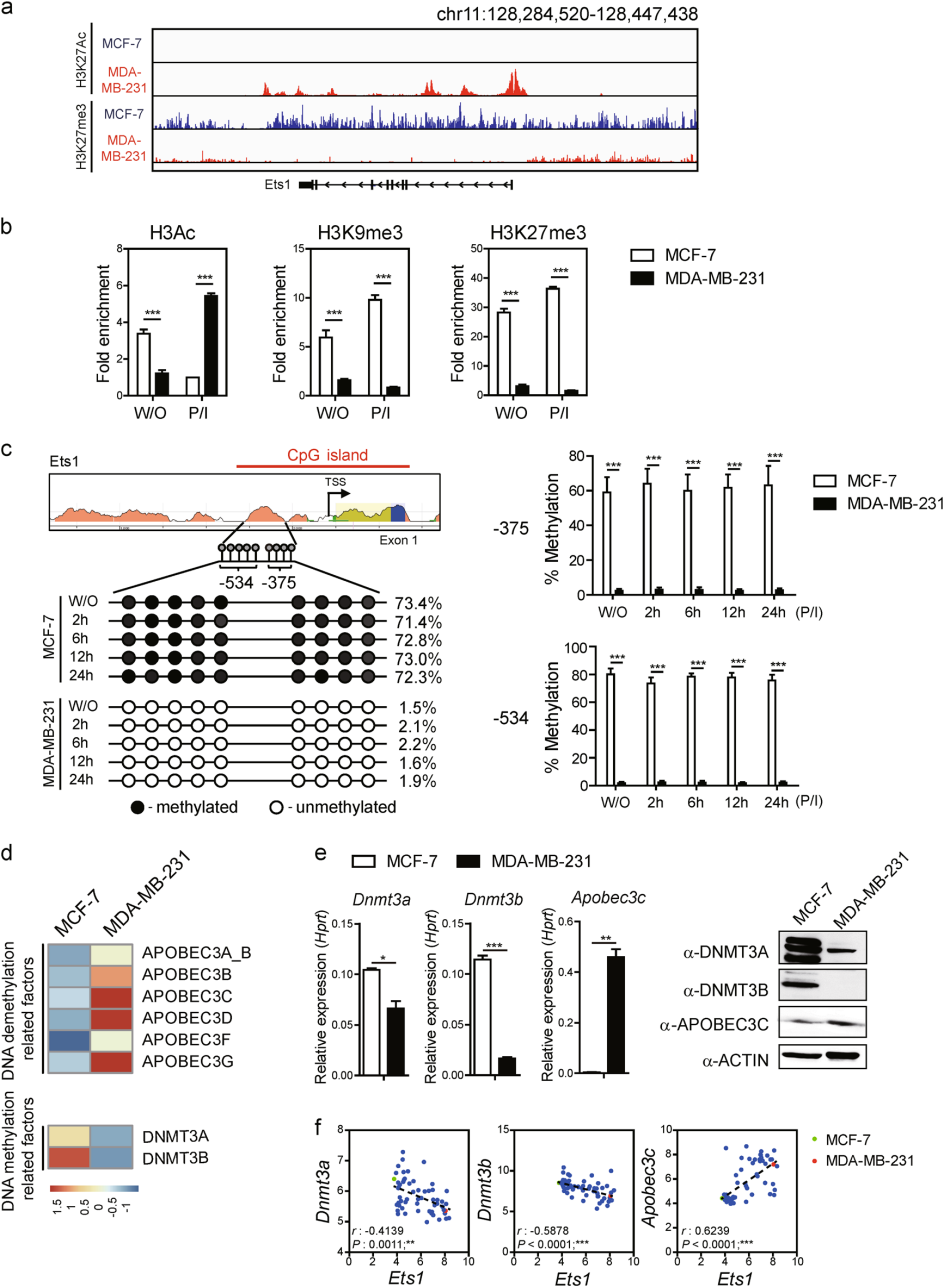
Close association of epigenetic status with Ets1 level. **a** ChIP-seq analysis of H3K27Ac and H3K27me3 at Ets1 promoter locus in MCF-7 (blue line) and MDA-MB-231 (red line) cells. **b** ChIP analysis of active marker (H3Ac) and inactive markers (H3K9me3 and H3K27me3) at Ets1 promoter locus under unstimulated or P/I stimulation conditions. The data from each replicate were normalized to the input control and the graphs represent fold enrichment of the indicated proteins to control antibody at the designated locus. Data are presented as mean ± SD. Two-way ANOVA with Bonferroni post-tests showed a significant difference of binding efficiency of H3Ac, H3K9me3, and H3K27me3 between MCF-7 and MDA-MB-231 cells. **c** DNA methylation status in the nine CpG sites (−375 or −534) was analyzed by bisulfite sequencing under unstimulated or stimulated (P/I) condition. Closed and open circles indicate methylated and unmethylated CpG sites, respectively. Bar graphs represent percentage methylation. Data are presented as mean ± SD. Two-way ANOVA with Bonferroni post-tests showed a significant difference of DNA methylation status. **d** Heatmap of RNA-sequencing data showing relative expression of DNA methylation-related and demethylation-related factors. **e** Analysis of relative expression levels in transcripts normalized against *Hprt* and proteins by qRT-PCR and Immuno-blots, respectively. Data are presented as mean ± SD. One-way ANOVA with Bonferroni correction indicated a significant difference of expression. **f** Scatterplots and nonparametric Spearman’s rank correlation (*ρ*) analysis with corresponding *p*-values correlation analysis from CCLE. Correlation between *Ets1* and DNA methylation-related genes (*Dnmt3a, Dnmt3b*, and *Apobec3c*) in mRNA. Each symbol represents an individual human breast cancer cell line. Green circle: MCF-7 cells; Red circle: MDA-MB-231 cells. **b**, **c**, **e** Data shown are representative of more than two independent experiments with similar results. *p < 0.05, **p < 0.01, and ***p < 0.001 (unpaired *t*-test)

Epigenetic abnormality in various cancer-related genes is often involved in enhanced tumorigenesis^33,34^. To compare the chromatin architecture between the MDA-MB-231 (Ets1^high^) and MCF-7 cells (Ets1^low^), we have performed chromatin accessibility by real-time PCR (CHART-PCR). Compared with MCF-7 cells (Ets1^low^), MDA-MB-231 (Ets1^high^) cells showed more open chromatin structure at the *Ets1* promoter region (Supplementary Figure S4a). Next we compared epigenetic status of histone modification and DNA methylation between the cells. First we analyzed ChIP-seq data set^35^ for H3K27Ac (active chromatin) and H3K27me3 (inactive chromatin) in the *Ets1* genomic locus between the cells. Compared to MCF-7 cells, MDA-MB-231 cells showed highly enriched H3K27Ac but decreased levels of H3K27me3 (Fig. 4a). By performing ChIP analysis, we confirmed that the *Ets1* locus in MDA-MB-231 cells is enriched for chromatin modifications associated with active gene transcription (H3Ac). On the other hand, chromatin modifications associated with repressed gene expression are under-represented (H3K9me3 and H3K27me3) (Fig. 4b).

Since DNA methylation is another epigenetic mechanism to control gene expression^36^, we compared DNA methylation status of individual CpG sites localized in CRE by bisulfide sequencing. All of nine CpG sites were highly methylated in MCF-7 but demethylated in MDA-MB-231 regardless of PMA/Ionomycin stimulation (Fig. 4c). We tested whether treatment of Decitabine (a DNA methyl-transferase inhibitor) or TSA (a HDAC inhibitor) could enhance Ets1 expression in MCF-7. Decitabine but not the TSA treatment enhanced Ets1 expression in MCF-7 (Supplementary Figures S4b, c). Moreover, Decitabine treatment increased invasiveness of MCF-7 cells and enhanced the binding efficiency of NFKB1 and RELA to CRE region (Supplementary Figures S4d, e). These results collectively suggest that chromatin architecture at the Ets1 promoter (CRE region) in MCF-7 cells is in a “less permissive” but not a “non-permissive” state.

To test whether differential DNA methylation status between the cells is due to changes in level of DNA methyl transferases, we analyzed a publicly available RNA-seq data set^37^. Compared with MDA-MB-231 cells, MCF-7 cells were found to express significantly higher levels of DNA methyl-transferase (*Dnmt3a* and *Dnmt3b*) but lower levels of APOBEC family genes (Fig. 4d, e). APOBEC is known as a cytidine deaminases and its dysregulation causes mutations in numerous cancer types. Furthermore, MCF-7 cells showed higher binding of DNMT3A and DNMT3B on CRE region (Supplementary Figure S5a) while MDA-MB-231 cells show no DNMT association to Ets1 locus due to their low expression (data not shown). We also tested whether levels of Ets1 expression could be altered upon overexpression of DNMT3A/3B or APOBEC3C in MDA-MB-231 and MCF-7 cells, respectively. Indeed, overexpression of DNMT3A/3B in MDA-MB-231 cells decreased Ets1 expression, whereas overexpression of APOBEC3C in MCF-7 cells slightly increased Ets1 expression (Supplementary Figures S5b, c). These results in part suggest availability of DNA methyl transferases can affect the level of Ets1 transcription.

We also questioned whether this finding could be generalized in various breast cancer cell lines (*n* = 59). Indeed, analysis of Cancer Cell Line Encyclopedia confirmed that *Ets1* expression was negatively correlated with *Dnmt3a* (Spearman *r* = −0.4139, **p = 0.0011) and *Dnmt3b* (Spearman *r* = −0.5878, ***p < 0.0001) but positively with *Apobec3c* (Spearman *r* = 0.6239, ***p < 0.0001) (Fig. 4f). Consistent with human cell line data, *Ets1* expression was negatively correlated with *Dnmt3a* and *Dnmt3b* in normal specimens, while *Ets1* level is positively correlated with *Apobec3c* in human breast cancer specimens (Supplementary Figure S5d). These results indicate that *Ets1* expression is epigenetically regulated in breast cancer.

### Deletion of CRE region reduces Ets1 expression and tumor invasiveness

**Fig. 5:**
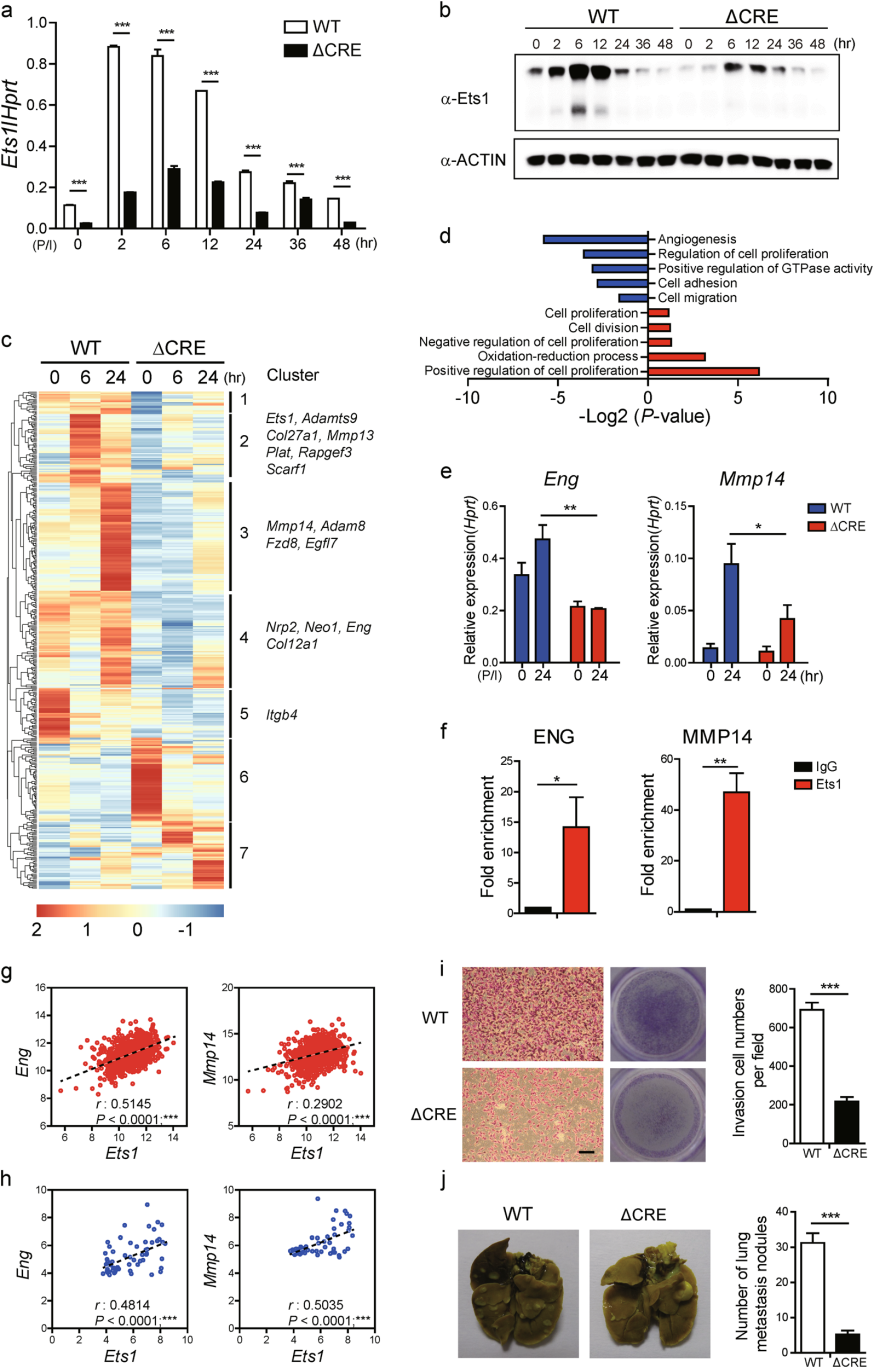
Deletion of CRE region reduces Ets1 expression and tumor invasiveness. **a**, **b** Comparative analysis of Ets1 expression levels in transcripts and protein between WT and CRE-deleted MDA-MB-231 (ΔCRE) cells determined by qRT-PCR and Immuno-blot. Transcript levels were normalized against *Hprt* and actin served as loading controls, respectively. Data are presented as mean ± SD. Two-way ANOVA with Bonferroni post-tests showed a significant difference of Ets1 expression. **c** Heatmap of RNA-sequencing data showing differentially expressed genes (DEGs) in WT and ΔCRE MDA-MB-231 cells. **d** GO-term enrichment using DAVID “Biological functions” category. Shown are the top five up and down-regulated functions based on –log2(*p* value). **e** Comparison of expression levels of Ets1 target genes between WT and ΔCRE cells. **f** ChIP-PCR was performed to detect Ets1 enrichment on the promoter regions of *Eng* and *Mmp14* genes in MDA-MB-231 cells 2 h after stimulation. Fold enrichment compared with input is shown. Data are representative of two individual experiments. Bars represent averages from triplicate PCR reactions as mean ± SD. One-way ANOVA with Bonferroni correction indicated a significant enrichment of Ets1; *p < 0.05; **p < 0.01; ***p < 0.001. **g** Scatterplots and nonparametric Spearman’s rank correlation analysis with corresponding *P*-values correlation analysis from TCGA. Correlation between *Ets1* and target genes (*Eng* and *Mmp14*) in mRNA levels. Each symbol represents an individual BRCA sample. **h** Scatterplots and nonparametric Spearman’s rank correlation analysis with corresponding *p*-values correlation analysis from CCLE. Correlation between *Ets1* and target genes (*Eng* and *Mmp14*) in mRNA levels. Each symbol represents an individual human breast cancer cell line. **i** Cells were stained with crystal violet and representative images were obtained from in vitro invasion assay using 10% FBS as chemoattractant. Cells were stained with crystal violet. Scale bar: 100 m. **j** Representative images (left) and numbers of metastasis nodules (right) of mouse lungs obtained from 6 weeks after tail vein injections of WT or ΔCRE cells (each 5 × 10^5^) into eight female nude mice, respectively. **i**, **j** Data are presented as mean ± SD. One-way ANOVA with Bonferroni correction indicated a significant difference between WT and ΔCRE cells. **a**, **b**, **e**, **f**, **i**, **j** Data shown are representative of more than two independent experiments with similar results. *p < 0.05, **p < 0.01, and ***p < 0.001 (unpaired *t*-test)

To validate the functional role of NFAT and NF-κB binding sites (−540bp to −270bp) within the CRE region (−540 to −80 from TSS) in Ets1-mediated tumorigenesis, we deleted the region by a CRISPR/Cas9 based KO system in metastatic MDA-MB-231 cells (Supplementary Figures S6a, b). Compared with wild-type MDA-MB-231 cells (WT), CRE-deleted cells (ΔCRE) showed lower levels of Ets1 expression in mRNA and protein (Fig. 5a, b). We then compared transcriptome profiles between WT and ΔCRE cells by RNA-seq analysis. ΔCRE cells showed altered expression of numerous genes (DEGs: *p*-value < 0.05; 0 h - Up: 285 and Down: 179, 6 h - Up: 627 and Down: 954, 24 h - Up: 544 and Down: 745) (Fig. 5c). Gene-annotation enrichment analysis (DAVID) showed that ΔCRE cells had alterations in cell adhesion and angiogenesis programs (Fig. 5d). Indeed, we found that ΔCRE cells express much lower level of *Eng* and *Mmp14* (Fig. 5e), critical factors for cell adhesion^38,39^. In addition, by performing ChIP assay, we confirmed the possibility that direct binding of Ets1 to the promoters of those target genes regulates expression levels (Fig. 5f). Analysis of human breast cancer specimens further confirmed this possibility. We analyzed 1000 human breast cancer specimens (Fig. 5g) and 59 human breast cancer cell lines (Fig. 5h) and found that levels of *Ets1* expression is well-correlated with *Eng* and *Mmp14* levels, accordingly (Fig. 5g, h).

To verify the functional role of CRE in Ets1-mediated metastasis of breast cancer cells, first we performed in vitro invasion assay. Compared with WT MDA-MB-231, ΔCRE cells showed significantly reduced invasive capacity (Fig. 5i). To further confirm whether CRE deletion reduces tumor invasiveness in vivo, nude mice were injected intravenously into the tail vein with either ΔCRE or WT MDA-MB-231 cells. After 6 weeks, tumor invasiveness was compared between the groups. Indeed, ΔCRE-injected mice showed a significant reduction of metastasis nodules in the lungs compared with mice injected with WT cells (Fig. 5j). Interestingly, Ets1 overexpression in ΔCRE cells significantly increased their invasive properties but was still less compared to the parent WT MDA-MB-231 cells (Supplementary Figures S7a, b). Moreover, overexpression of both NFATc2 and NFKB1/RELA into ΔCRE cells increased Ets1 expression and their invasive properties, but was still less compared to the WT MDA-MB-231 cells (Supplementary Figures S7c, d). Collectively, these data demonstrate a critical role of CRE in *Ets1* gene expression and Ets1-mediated metastasis of breast cancer cells.

## Discussion

In this study, we have found that *Ets1* gene expression is regulated epigenetically as well as by the crosstalk between NFATc2/NF-κBs and core-regulatory element (CRE) located in the Ets1 promoter. Deletion of CRE region down-regulated Ets1 expression and, consequently, reduced breast cancer progression by altering cancer-associated genetic programs.

Ets1 expression and function are regulated by diverse mechanisms. Previous studies reported that *Ets1* gene expression is transcriptionally regulated by several transcription factors including Ets1, AP-1, AP-2, and Oct in human T cell and B cell lines^24,40,41^. In this study, we identified the core-regulatory element (CRE) region in the Ets1 promoter that contains binding sequences for NFATs and NF-κBs. Binding of NFATc2 and NFKB1/RELA heterodimer to the CRE region synergistically contribute to the expression of *Ets1*. NFATs and NF-κBs factors are reported as downstream targets of calcium and PKC signaling, respectively, and affect expression level of their target genes associated with tumor pathogenesis^42–47^. Our finding support the previous reports that calcium signaling affects multiple cellular responses in the tumor microenvironment^48,49^. Ca^2+^-NFAT signaling is associated with growth and development of malignant breast cancer^43^. Ca^2+^ ionophore, ionomycin synergizes with PMA in enhancing the activation of PKC^50^. In accordance with this notion, we observed that *Ets1* expression was synergistically increased upon PMA/Ionomycin (P/I) stimulation (Fig.1f).

Physical binding of transcription factors to the regulatory region requires permissive chromatin architecture. Moreover, epigenetic changes including aberrant DNA methylation are tightly linked to cancer development^51^. For example, DNMT3A mutation significantly reduced methylated DNA levels and reduced survival rate in AML patients by causing enhanced expression of HOXB genes^52^. Furthermore, alteration in APOBEC expression level also caused increased frequencies of TP53 mutation derived-inactivation in breast cancer development^53^. Although Activation-induced cytidine deaminase (AID)/APOBEC cytosine deaminases have been shown to implicate in active DNA demethylation^54^, it is not clear whether aberrant DNA demethylation causes uncontrolled gene regulation in cancer development. We found that metastatic MDA-MB-231 (Ets1^high^) cancer cells have active chromatin status in the *Ets1* locus compared to less metastatic MCF-7 (Ets1^low^) cells. These findings suggested that changes in level of epigenetic regulators regulate Ets1 expression. DNA methylation and demethylation are mediated by DNA methyl-transferase enzymes (DNMT1, 3A, and 3B) and TET enzymes (TET1, 2, and 3)/DNA deaminase (AID/APOBEC family), respectively. In depth analysis of gene expression profiles showed that indeed, MCF-7 (Ets1^low^) cells express much higher levels of DNA methyl-transferase such as *Dnmt3a* and *Dnmt3b*, while *Apobec3c* levels were increased (Fig. 4d, e). Based on these findings, we believe that levels of DNMT3A and DNMT3B regulate methylated status of CRE region, leading to differential Ets1 expression between metastatic versus less metastatic cells.

Deletion of NFAT and NF-κB binding sites (−540 bp to −270 bp from TSS) within the CRE region (−540 to −80) in metastatic breast cancer cells (ΔCRE cells) diminished Ets1 levels and reduced expression levels of invasion related genes. Gene ontology analysis shows that ΔCRE cells showed a significant alteration in the levels of ‘cell adhesion’ and ‘angiogenesis’ such as *Nrp2*, *Pik3cg*, *Mmp14*, *Eng*, *Adam8* and *Adam15* (Fig. 5c, d). In particular, we found that Ets1 directly regulates *Eng* and *Mmp14* expression by binding to their cis-regulatory regions (Fig. 5e, f). *Eng* encodes Endoglin/CD105, a co-receptor for transforming growth factor-β (TGF-β) family. Endoglin (ENG) is known as a critical factor for tumor invasion by enhancing formation of invadopodia, extracellular proteolysis, chemotaxis, and migration^55^. Elevated ENG in breast tumor tissue is associated with higher metastatic risk and poor prognosis^56,57^. In addition, ΔCRE cells showed a significant alteration in the levels of matrix metalloproteinase MMP14 that has an important role in tumor invasion by regulating the levels of collagens, extracellular matrix (ECM) proteins and factors involved in epithelial–mesenchymal transition^38,58,59^. These findings suggest that binding of NFATc2 and NFKB1/RELA to the CRE might be a key mechanism for enhanced Ets1 expression and increased invasive properties of metastatic breast cancers. However, we could not rule out other possibilities involved in this process. Indeed, overexpression of NFATc2, NFKB1/RELA in ΔCRE cells partially restored Ets1 expression and invasiveness (Supplementary Figures. S7c, d). These results suggest that within the CRE region, other region in the Ets1 promoter may also contain responsive sequences for NFATc2, NFKB1/RELA. Indeed, we found conserved NFATs/NFkB binding motifs encompassing −80/−270bp region. Reporter assay showed this region has promoter activity, which was enhanced upon stimulation (Fig. 2a). Another possibility is that NFATc2 and NFKB1/RELA may regulate invasiveness of cancer cells in an Ets1-independent manner. Indeed, NFATs are known to promote invasion by inducing Cox2 expression^60^. NFKB also affects invasiveness of breast cancer cells by regulating CD44 level^61^.

In summary, our study suggests that the crosstalk between NFATc2, NFKB1/RELA, and CRE region regulates *Ets1* gene transcription in metastatic breast cancer cells. Inhibition of this interaction could be a therapeutic approach to reduce Ets1 levels and Ets1-mediated tumor invasiveness of breast cancer cells.

## Materials and methods

### Cell culture, plasmid, and reagents

Cells were cultured in DMEM (WELGENE: LM 001–05) supplemented with 10% FBS (Gibco: 10099–141) and 100 U/ml of penicillin–streptomycin (Thermo: 15140122). Cells were harvested with 0.05% trypsin-EDTA (Gibco: 25300–054). Ets1 promoter region (hEts1, 1500 bps) was amplified with cDNA from MDA-MB-231 cells and inserted into pXPG vector. From this template, different deletion constructs were prepared by PCR and cloned into pXPG vector as shown in Fig. 2a. NFAT and/or NF-κB binding sites in Ets1 promoter were mutated by QuikChange XL Site Directed Mutagenesis Kit (Agilent: #200517). hEts1 promoter-ΔNFAT-Luc, or ΔNF-κB-Luc were constructed by introducing mutations at the consensus NFAT site (TTTTAA → TTCCCGA), or NF-κB site (TTGGAA → TTAAGGA), respectively. All sequences were verified by Sanger sequencing. The following chemicals were used; phorbol 12-myristate 13-acetate (PMA, Calbiochem: 524400), Ionomycin (Calbiochem: 407950), Cyclosporin A (Calbiochem: 239835), Decitabine (Selleckchem: 2353–33–5), and Trichostatin A (Selleckchem: 58880–19–6). All plasmids were obtained from Addgene (WT-NFAT1: #11100, pREP-NFAT2: #11788, pREP-NFAT3: #11789, pREP-NFAT4: #11790, pEGFP NFAT5: #13627, pCMV4-p50: #21965, pCMV4-p52: #23289, pcDNA-FLAG-REL: #27253, GFP-RelA: #23255, pcDNA3/Myc-DNMT3A: #35521, pcDNA3/Myc-DNMT3B1: #35522 and pcDNA3.1 human A3C: #105047).

### RNA isolation, cDNA synthesis, and quantitative RT-PCR (qRT-PCR)

Total RNA was extracted using TRI Reagent (Molecular Research Center, Ohio) following standard protocols. Reverse transcription of 1.0ug of RNA was performed using oligo (dT) primer (Promega: C1101) with Improm II Reverse Transcription system (Promega, Madison, WI) according to the manufacturer’s protocol. Quantitative RT-PCR was performed using SYBR Green Dye mix (Takara: RR420) on Rotor-Gene Q (Qiagen, Hilden). Data were normalized to human hypoxanthine-guanine phosphoribosyl transferase (HPRT). Primer sequences are provided in Supplementary Table 1.

### Luciferase reporter assays

Luciferase reporter plasmids containing various regions of Ets1 promoter were co-transfected with indicated expression plasmids (such as NFATc2, NFKB1, RELA, AP-1, and Ets1) in MDA-MB-231 cells using Gene Expresso (Excellgen: EG-1086) according to the manufacturer’s protocol. Renilla luciferase was co-transfected as an internal control for transfection. After 24 h of transfection, cells were harvested and luciferase activity was measured by the dual luciferase assay system (Promega: E1910) similar to a previous study^62^.

### Nuclear extraction and immuno-blot

Whole cell lysates were extracted using RIPA buffer and nuclear/cytoplasmic fractions were isolated using nuclear and cytoplasmic extraction kit (Thermo: 78833) according to the manufacturer’s protocol. Protein concentration was measured by Bradford protein assay (Bio-Rad: #5000001) and 20 μg or 30 μg of proteins were used for SDS-PAGE (10%), and then transferred onto a nitrocellulose membrane (Bio-Rad: 162–0097). The following primary antibodies were used from Abcam (Cambridge, UK): anti-NFATc2 (ab2722), anti-NFKB1 (ab7971), anti-RELA (ab7970), anti-DNMT3A (ab13888), anti-DNMT3B (ab13604) and anti-ACTIN (ab3280); Santa Cruz Biotechnology (Santa Cruz, CA, USA): anti-Ets1 (sc-350); Cell signaling Technology (Danvers, MA, USA): LaminB1 (#12586); GeneTex (Irvine, TX, USA): APOBEC3C (GTX102164). Protein expression was visualized with an ImageQuant™ LAS 4000 (GE healthcare Life Science, Piscataway, NJ). Actin and LaminB1 expression were measured as loading controls for whole-cell lysates, and nuclear protein, respectively. Band intensity was quantified using ImageJ software (NIH).

### Transfection of expression vectors and siRNAs

siRNA-mediated depletion was accomplished using Scrambled negative control (SR30004), siRNAs for NFATc2 (SR303150), NFKB1 (SR303161) and RELA (SR304030) purchased from Origene (OriGene Technologies, Rockville, MD) using lipofectamine RNAiMAX reagent (Invitrogen: 13778–100) according to the manufacturer’s protocol. After 48 h, transfected cells were used for the further experiments. Plasmids for NFATc2, NFKB1, and RELA were transfected into MCF-7 cells using Gene Expresso (Excellgen: EG-1086) according to the manufacturer’s protocol.

### Chromatin immunoprecipitation (ChIP)-PCR assay

ChIP-PCR assays were performed by Simple ChIP plus Enzymatic Chromatin IP Kit (Cell Signaling: #9005) according to the manufacturer’s protocol. Briefly, cells were cross-linked with 1% of formaldehyde and lysed for nuclei preparation. Nucleus pellet was treated with Micrococcal nuclease and sonicated for chromatin fragmentation. Protein–chromatin complex was incubated with antibodies targeting anti-NFATc2 (ab2722), anti-NFKB1 (ab7971), anti-RELA (ab7970), anti-DNMT3A (ab13888), anti-DNMT3B (ab13604), anti-H3K4me1 (ab8895), anti-H3K4me3 (ab8580), anti-H3K9me3 (ab8898), anti-Ets1 (Cell Signaling: #14069), anti-H3Ac (Merck: 06–599), or anti-H3K27me3 (Merck: 07–449) at 4 °C for overnight. Rabbit or mouse IgG (Vector Laboratories) was used as negative control. After immunoprecipitation, 50 μl of Dynabeads protein G or A (Life technologies, Oslo) were added and rotated further for 6 h at 4 °C. Ab/protein/chromatin complexes were reverse-cross-linked at 65 °C overnight and DNA was purified by DNA purification columns (Cell Signaling: #10010). The relative enrichment of specific regions of precipitated DNA were measured by real-time PCR (qPCR). To quantify protein binding in specific genomic locus, purified DNA was used for real-time PCR. Primer sequences are listed in Supplementary Table 2. H3K27Ac, and H3K27me3 ChIP-seq data set (GSE38548) were re-analyzed^35^.

### Bisulfite treatment and pyrosequencing

DNA methylation analysis was performed by bisulfite pyrosequencing. Briefly, genomic DNA (200 ng) was subjected to bisulfite treatment with EZ DNA methylation-Lightning Kit (Zymo Research: D5030). Bisulfite-treated DNA was amplified using specific primers (Supplementary Table 3) with PCR premix (Enzynomics, Korea); PCR conditions: denaturing at 95 °C for 10 min, followed by 45 cycles at 95 °C for 30 s, at 55 °C for 30 s, at 72 °C for 30 s, and a final extension at 72 °C for 5 min. Subsequently, sequencing was performed on a PyroMark ID system (Qiagen) with the Pyro Gold reagent kit (Qiagen: #40–0045). Unmethylated/Methylated control DNA (Qiagen: #59568/59655) was used as a negative or positive control.

### Chromatin accessibility analysis

Chromatin accessibility was analyzed by EpiQuik Chromatin Accessibility Assay Kit (Epigentek: #P-1047) according to the manufacturer’s protocol. Briefly, chromatin from MCF-7 or MDA-MB-231 cells was treated with a nuclease (Nse) mix for 2 min. Nse-treated and No-Nse control DNA were amplified by quantitative PCR with primers targeting specific region on Ets1 promoter (Supplementary Table 2). Fold enrichment (FE) was calculated by using the formula FE = 2 ^(Nse CT-no-Nse CT)^ 100%.

### Deletion of CRE region by CRISPR/Cas9 based KO system

To delete the NFAT and NF-κB binding sites (−540 bp to −270 bp from TSS) within the core-regulatory element (CRE; −540 to −80), guide RNA (gRNA) was designed and mismatch sensitive nuclease assay (T7E1 assay) was performed for gRNA screening as described previously^63^. After screening gRNA, MDA-MB-231 cells were transiently transfected with Cas9 plasmid, gRNA F and gRNA R with Lipofectamine 2000 (Invitrogen). gRNA sequence were as follows: F: 5′-ACGCAGGAGCATTACATGGGTGG-3′; R: 5′-GGAGCAGTGCGTGGAGCCCC GGG-3′. After 48 h of transfection, target deleted clones were screened *via* genomic DNA analysis. Genomic DNA was analyzed by PCR and DNA sequencing for selecting promoter deleted cells. The primer pairs used for PCR analysis were F: 5′-CAAAGCGAAAGGAAGGGCTG-3′; R: 5′-GGAGGTAAATTGGAAGCTTACGG-3′. Mutations were confirmed by Sanger sequencing and the effect of CRE deletion on Ets1 level was tested by immuno-blot.

### In vitro invasion assay and analysis of lung metastasis in vivo

For in vitro invasiveness assay, trans-well chamber (8 m pore size; Corning: 354578) and Matrigel (Corning: 354234)-coated membrane were used. Briefly, cells (2.5 × 10^4^) were seeded in upper chamber containing serum-free medium and serum-containing medium in lower chamber as a chemoattractant. After 12 h of incubation, cells on the lower surface of the membrane were fixed and stained with the 0.5% crystal violet stain solution to calculate total number of invaded cells. For lung metastasis assay in vivo, WT or ΔCRE MDA-MB-231 cells (1 × 10^6^) were intravenously injected into tail vein of 7-week-old nude mice. No blinding test was done in this study. The injected mice were killed after 6 weeks to analyze metastasis in lung. The lungs were fixed in picric acid overnight and the tumor colonies were counted under a dissection microscope. Animal experiments were performed in accordance with the guide-lines and regulations, and with the approval of Institutional Animal Care and Use Committee (IACUC) at POSTECH.

### RNA-sequencing

RNA-sequencing was performed by NextSeq 500 Sequencing System. Total RNA was extracted and purified with RibospinTMII (GeneAll biotechnology: 314–150). RNA was subjected to library preparation with TruSeq Stranded mRNA Sample Preparation Kit (Illumina: RS-122–2101~2) and RNA-sequencing was performed by NextSeq 500 Sequencing System (Illumina). Sequences were mapped to hg19 with TopHat (version 2.0.12). Estimated expression level was generated with Cufflinks (version 2.2.1) and differentially expressed genes were selected using Cuffdiff (version 2.2.1). RNA-seq was performed on two biological replicates. The set of differential expressed genes was analyzed using DAVID gene functional classification tool^64^. For analysis of gene expression between MCF-7 and MDA-MB-231 cells shown in Fig. 4d, raw data from the NCBI database (GSE48213) were analyzed^65^.

### Analysis of data set for human samples and cell lines

Data of human breast tumor specimens (*n* = 1100) were obtained from The Cancer Genome Atlas (TCGA) database (https://tcga-data.nci.nih.gov/tcga/). mRNA expression data (RNA Seq V2 RSEM) were obtained through cBioPortal (http://www.cbioportal.org/). The Human breast cell lines (*n* = 59) expression data were obtained from Cancer Cell Line Encyclopedia (https://portals.broadinstitute.org/ccle). Spearman correlation coefficients analysis was used to evaluate the gene expression correlation.

### Statistical analysis

Statistical analysis of at least three independent experiments was performed. Error bars indicates standard deviation (SD). All student *t*-tests performed were student two-tailed tests (*p < 0.05, **p < 0.01, and ***p < 0.001). For comparisons of more than two groups, data were analyzed using one-way analysis of variance (ANOVA) and adjusted by the correction of Bonferroni. For data with more than one independent variable, two-way ANOVA was used. Data were calculated and graphed with GraphPad Prism software.

### Data accessibility

RNA-seq data sets have been deposited in the GEO database with accession code GSE106634

## Electronic supplementary material

Supplementary Figures and Tables
