# Supplementary material for: Upregulation of Ets1 expression by NFATc2 and NFKB1/RELA promotes breast cancer cell invasiveness
Source: Oncogenesis. 2018 Nov 23;7(11):91. doi: 10.1038/s41389-018-0101-3 (PMC6250664; doi:10.1038/s41389-018-0101-3)
Supplement: Supplementary file 1 — Supplementary Figures and Tables [file 41389_2018_101_MOESM1_ESM.pdf]

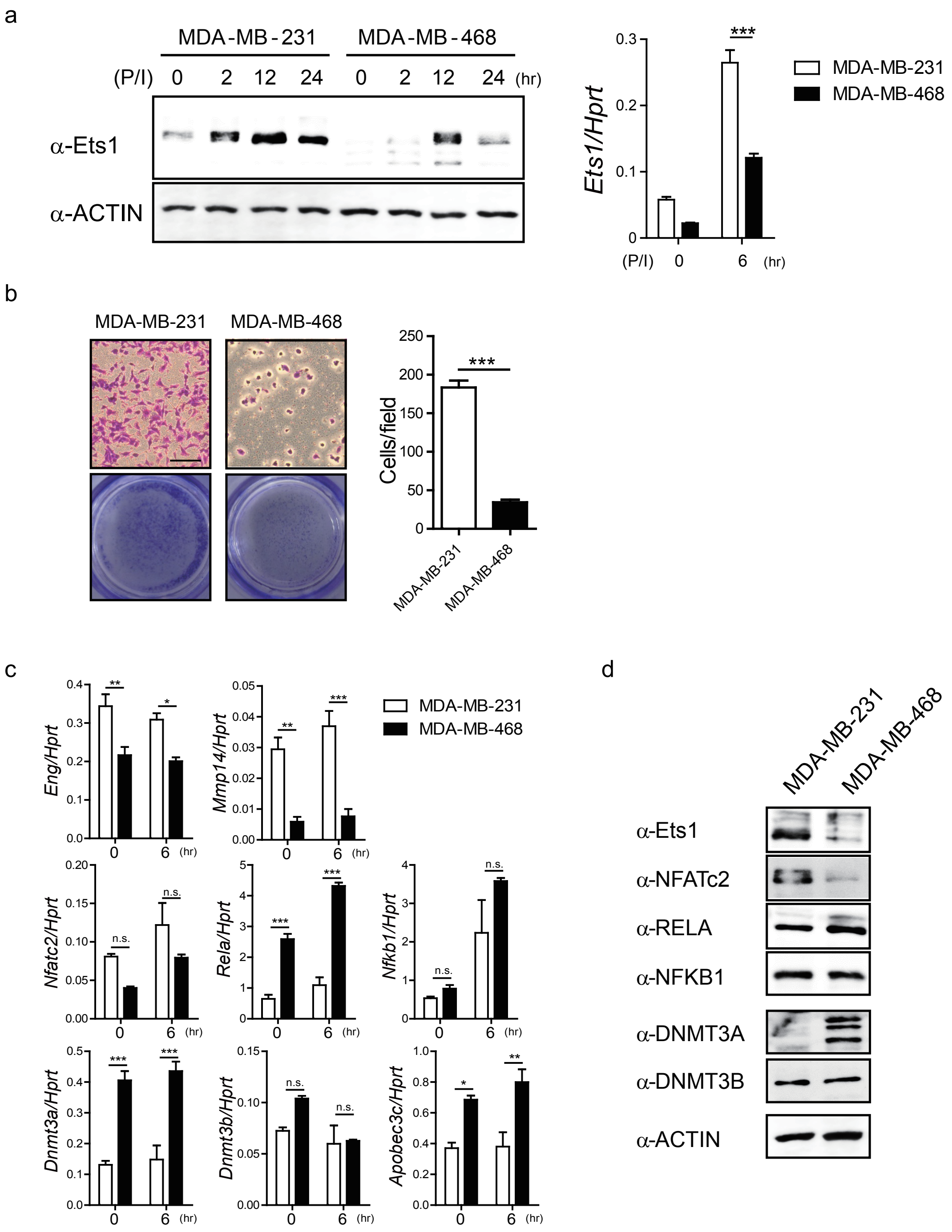

Supplementary Figure 1. Comparative analyses of Ets1 expression between metastatic MDA-MB-231 and non-metastatic MDA-MB-468 breast cancer cells. (a) Analyses of Ets1 protein and transcripts levels by Immuno-blot and qRT-PCR. (b) Cells were stained with crystal violet and representative images were obtained from *in vitro* invasion assay using 10% FBS as chemoattractant. Scale bar: 100μm. (c-d) Comparison of trans-acting factors of Ets1 and Ets1 target genes transcript and protein levels in MDA-MB-231 and MDA-MB-468 cells determined by (c) qRT-PCR normalized against *Hprt* and (d) Immune-blot. (a-c) Data shown are representative of more than three independent experiments with similar results. n.s.; not significant. *P < 0.05, **P < 0.01 and ***P < 0.001

Supplementary Fig 2. Kim et al.

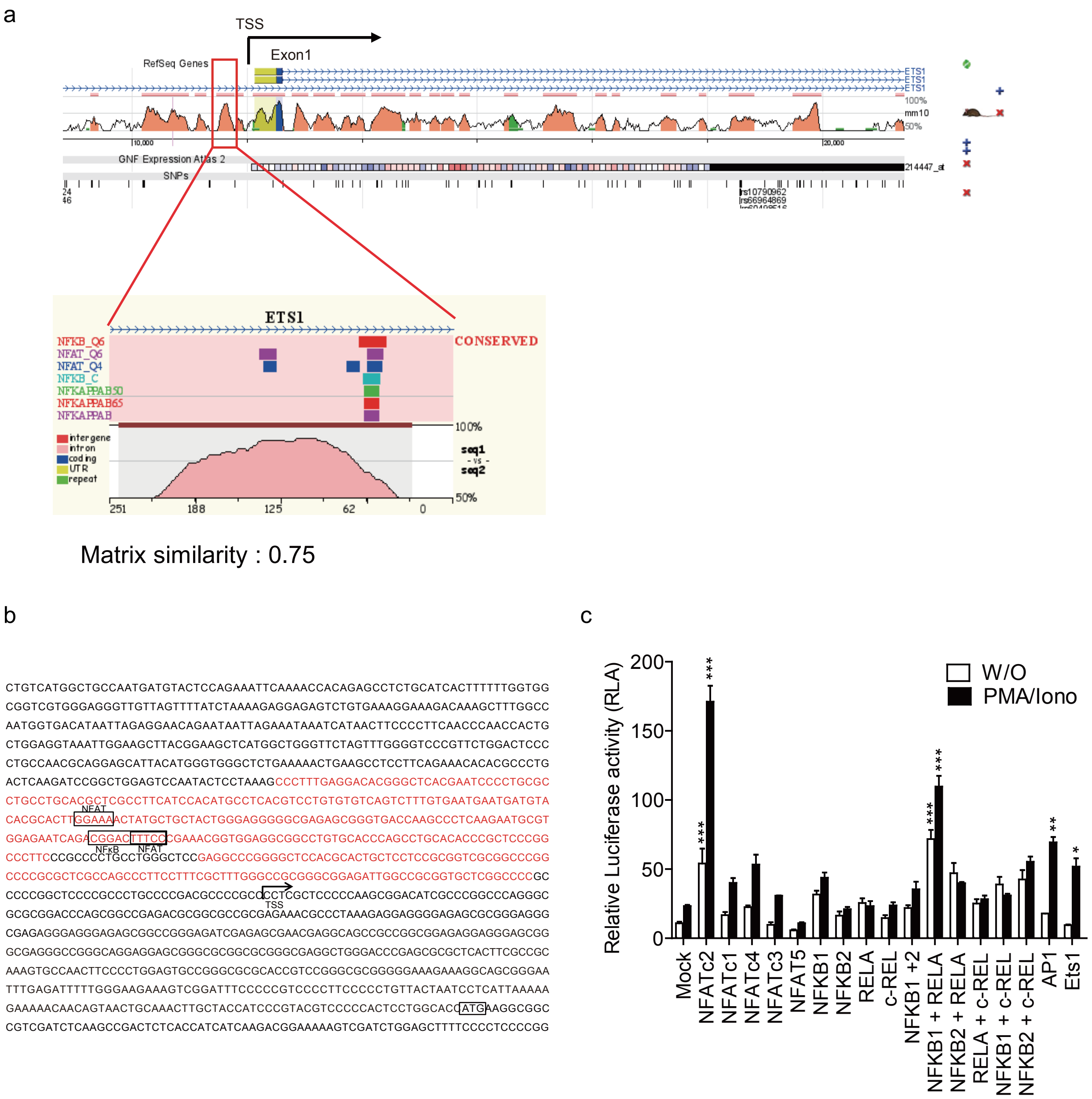

Supplementary Figure 2. *In silico* analysis of transcription factor binding sites on the Ets1 promoter region. (a) ECR browser analysis of the human and murine *Ets1* gene loci is shown. The red boxed region indicates the putative promoter region of the *Ets1* gene. The mouse genomic sequence is used as the base sequence on the x-axis. The boxed region indicates shared NFATs and NFKBs binding sites between human and mouse (matrix similarity: 0.75). (b) 5'-end genomic sequence of human *Ets1* gene locus with transcription start site (TSS) indicated in black arrow. Translation start site (ATG) and putative binding sites for transcription factors (NFAT and NFKB) are indicated and boxed. (c) Luciferase reporter assays were performed in the MDA-MB-231 cells transfected with Ets1 promoter-Luc reporter vector (540bp) and together with indicated combination of expression vectors. Relative luciferase activity in response to PMA/Ionomycin stimulation was measured. Data shown are representative of three independent experiments with similar results. *p < 0.05, **p < 0.01 and ***p < 0.001.

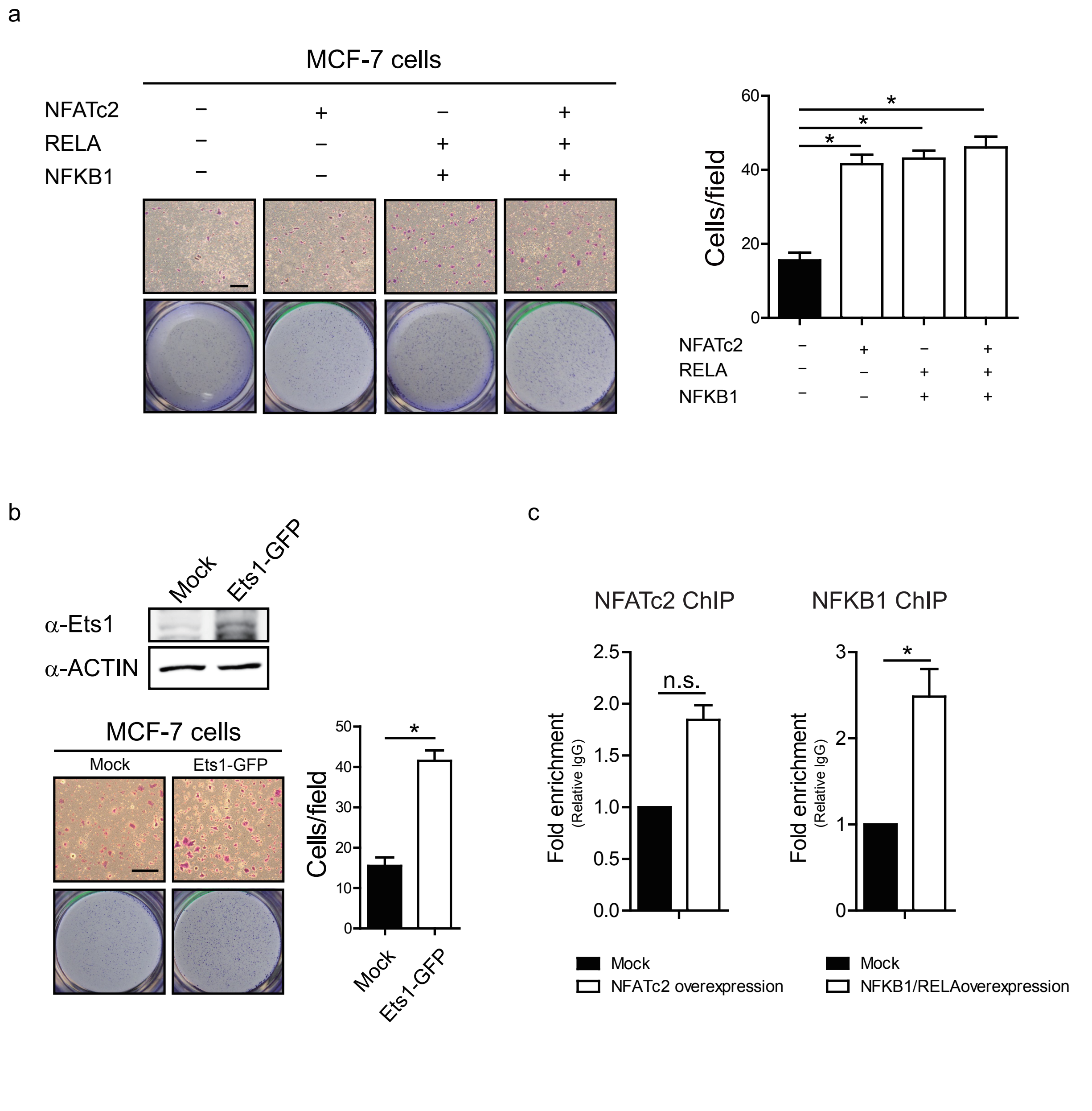

Supplementary Figure 3. Overexpression of NFATc2, NFKB1/RELA and Ets1 enhances invasion properties of MCF-7 cells. (a,b) Invasion assay was performed in MCF-7 cells. Cells were transfected with mock or indicated expression vectors individually or in combination. Scale bar: 100μm. (c) MCF-7 cells were transfected with mock or indicated expression vectors, then enrichment of NFATc2 and NFKB1 at Ets1 promoter locus was analyzed ChIP assay. The data from each replicate were normalized to the input control and the graphs represent fold enrichment of the indicated proteins to control antibody at the designated locus. (a-c) Data shown are representative of more than three independent experiments with similar results. n.s.; not significant. *P < 0.05

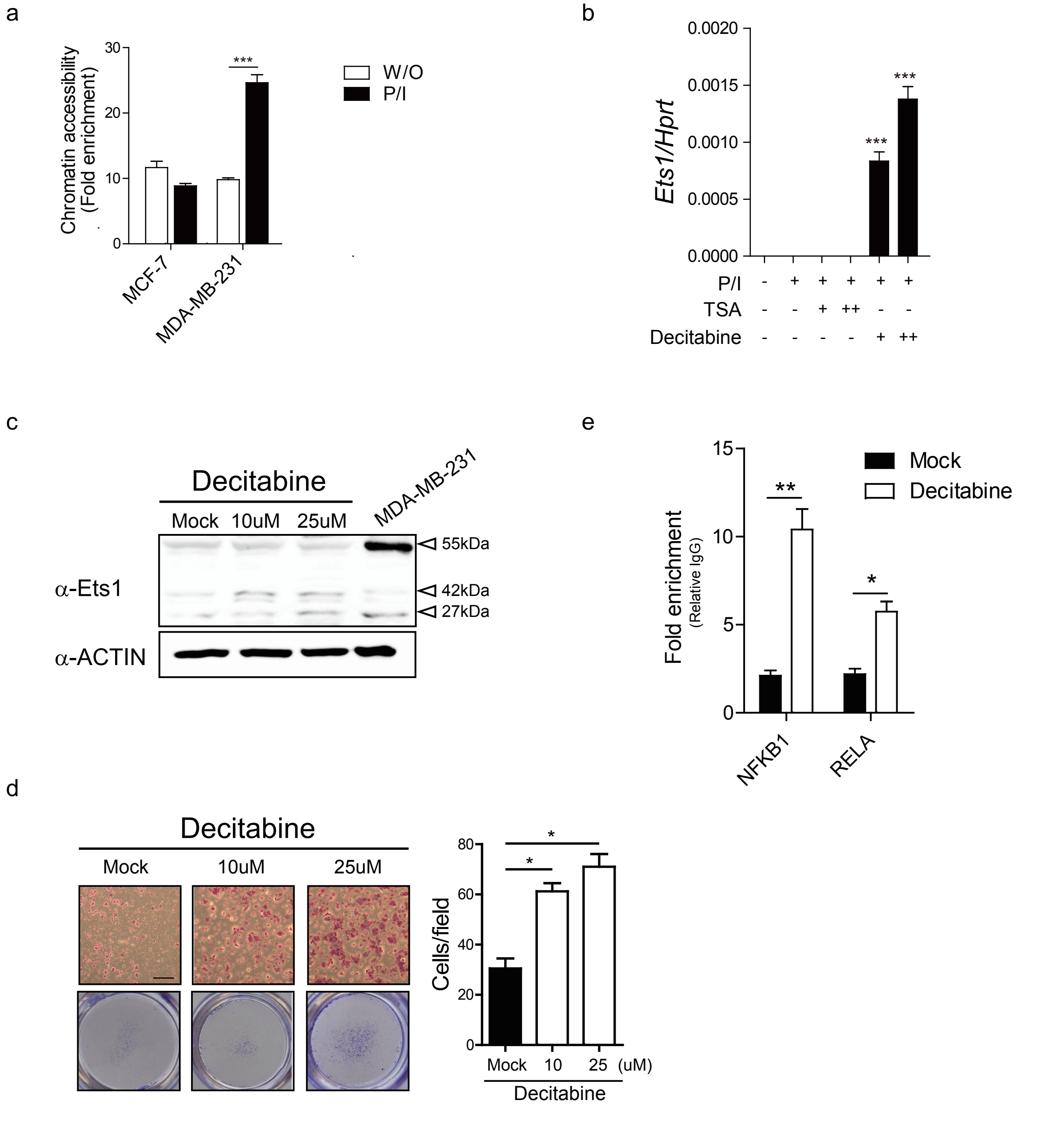

Supplementary Figure 4. Decitabine induces Ets1 expression and invasive properties of MCF-7 cells. (a) Nuclei isolated from the cells left untreated or stimulated with PMA/Ionomycin (P/I) were treated with Nuclease mixture as described in Materials and Methods. Chromatin accessibility by real-time PCR (CHART-PCR) was performed at the Ets1 promoter in MCF-7 and MDA-MB-231 cells. (b) Effect of Decitabine (DNA methyltransferase inhibitor) and TSA (histone acetylation inhibitor) treatment in MCF-7 cells on Ets1 transcript level determined by qRT-PCR normalized against *Hprt*. (c) Analyses of Ets1 protein levels by Immuno-blot. (d) Invasion assay after Decitabine (Mock, 10 and 25uM) treatment in MCF-7 cells. Scale bar: 100μm. (e) After Decitabine treatment, relative enrichment of NFKB1 and RELA to the CRE region determined by ChIP analysis. (a-c) Data shown are representative of more than three independent experiments with similar results. *P < 0.05, **P < 0.01 and ***P < 0.001

Supplementary Fig 5. Kim et al.

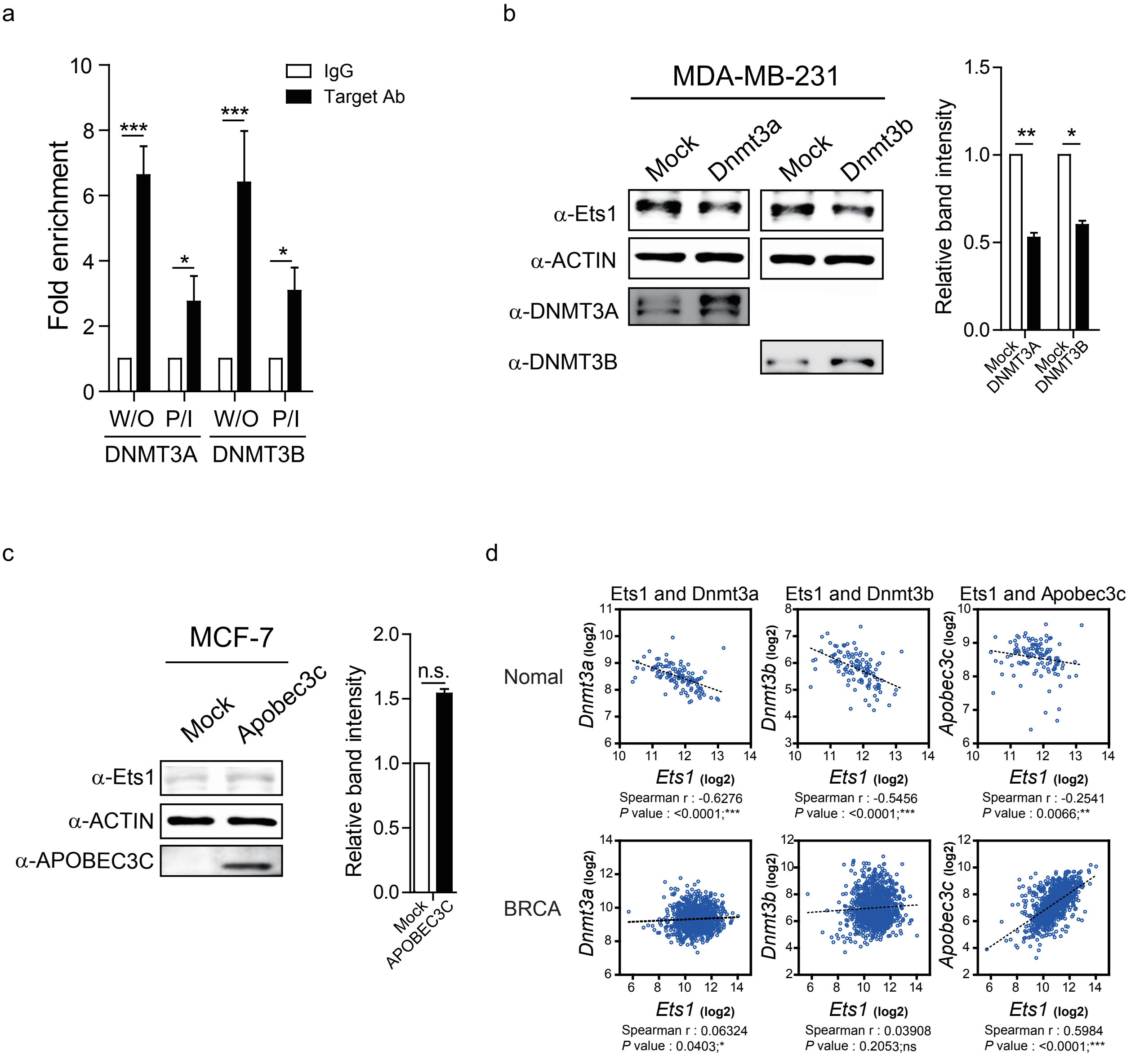

Supplementary Figure 5. DNMT3A/3B and APOBEC3C regulate Ets1 expression through binding to Ets1 promoter loci. (a) Chromatin immunoprecipitation analysis of DNMT3A and DNMT3 at *Ets1* promoter locus in MCF-7 cells under unstimulated and P/I stimulation conditions. The data from each replicate were normalized to the input control and the graphs represent fold enrichment of the indicated proteins to control antibody at the designated locus. (b-c) Analyses of Ets1 protein levels by Immuno-blot after overexpression of *Dnmt3a/3b* (b) and *Apobec3c* (c) in MDA-MB-231 and MCF-7 cells, respectively. Relative band intensity of Immuno-blot (b-c) was quantified by Image J software. (d) Scatterplots and Spearman's rank correlation from TCGA database. Correlation of *Ets1* with *Dnmt3a/3b* and *Apobec3c* in normal and BRCA mRNA. Each symbol represents an individual human specimen. (a,c,d) Data shown are representative of three independent experiments with similar results. n.s.; not significant. *P < 0.05, **P < 0.01 and ***P < 0.001

Supplementary Fig 6. Kim et al.

a

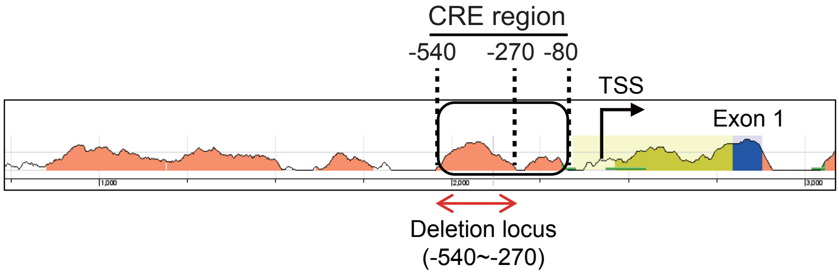

Target Sequence

guide RNA_F

CTGGAGGTAAATTGGAAGCTTACGGAAGCTCATGGCTGGGTTCTAGTTTGGGGTCCCGTTCTGGACTCCCCTGCCAACGCAGGAGCATTACATGGGTGGGC
TCTGAAAAACTGAAGCCTCCTTCAGAAACACACGCCCTGACTCAAGATCCGGCTGGAGTCCAATACTCCTAAAG*CCCTTTGAGGACACGGGCTCACGAATCC*
CCTGCGCCTGCCTGCACGCTCGCCTTCATCCACATGCCTCACGTCCTGTGTGTCAGTCTTTGTGAATGAATGATGTACACGCACTTGGAAAACTATGCTGCT
ACTGGGAGGGGGCGAGAGCGGGTGACCAAGCCCTCAAGAATGCGTGGAGAATCAGACGGACTTTCCCGAAACGGTGGAGGCGGCCTGTGCACCCAGCCT
*GCACACCCGCTCCCGGCCCTTC*CCGCCCCTGCCTGGGCTCCGAGGCCCGGGGCTCCACGCACTGCTCCTCCGCGGTCGCGGCCCGGCCCCGCGCTCGC
guide RNA_R
CAGCCCTTCCTTTCGCTTTGGGCCGCGGGCGGAGATTGGCCGCGGTGCTCGGCCCCGCCCCCGGCTCCCCGCCCTGCCCCGACGCCCCGCCCCTCGCTC
CCCCAAGCGGACATCGCCCGGCCCAGGGCGCGCGGACCCAGCGGCCGAGACGCGGCGCCGCGAGAAACGCCC

b

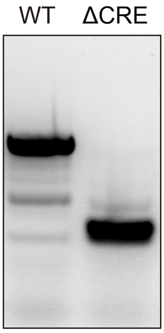

Supplementary Figure 6. Generation of CRE region-deleted (ΔCRE) MDA-MB-231 cells. (a) Schematic depiction of the CRE region (boxed in black) in the Ets1 promoter locus. The sequence of target sites for deletion (-540 to -270bp) within the CRE marked in red color, and for guide RNAs (gRNA) forward- and reverse-sites indicated in yellow and green color, respectively. (b) Confirmation of CRE deletion by genotyping of WT and ΔCRE MDA-MB-231 cells by PCR with primers shown in the Materials and Methods.

Supplementary Fig 7. Kim et al.

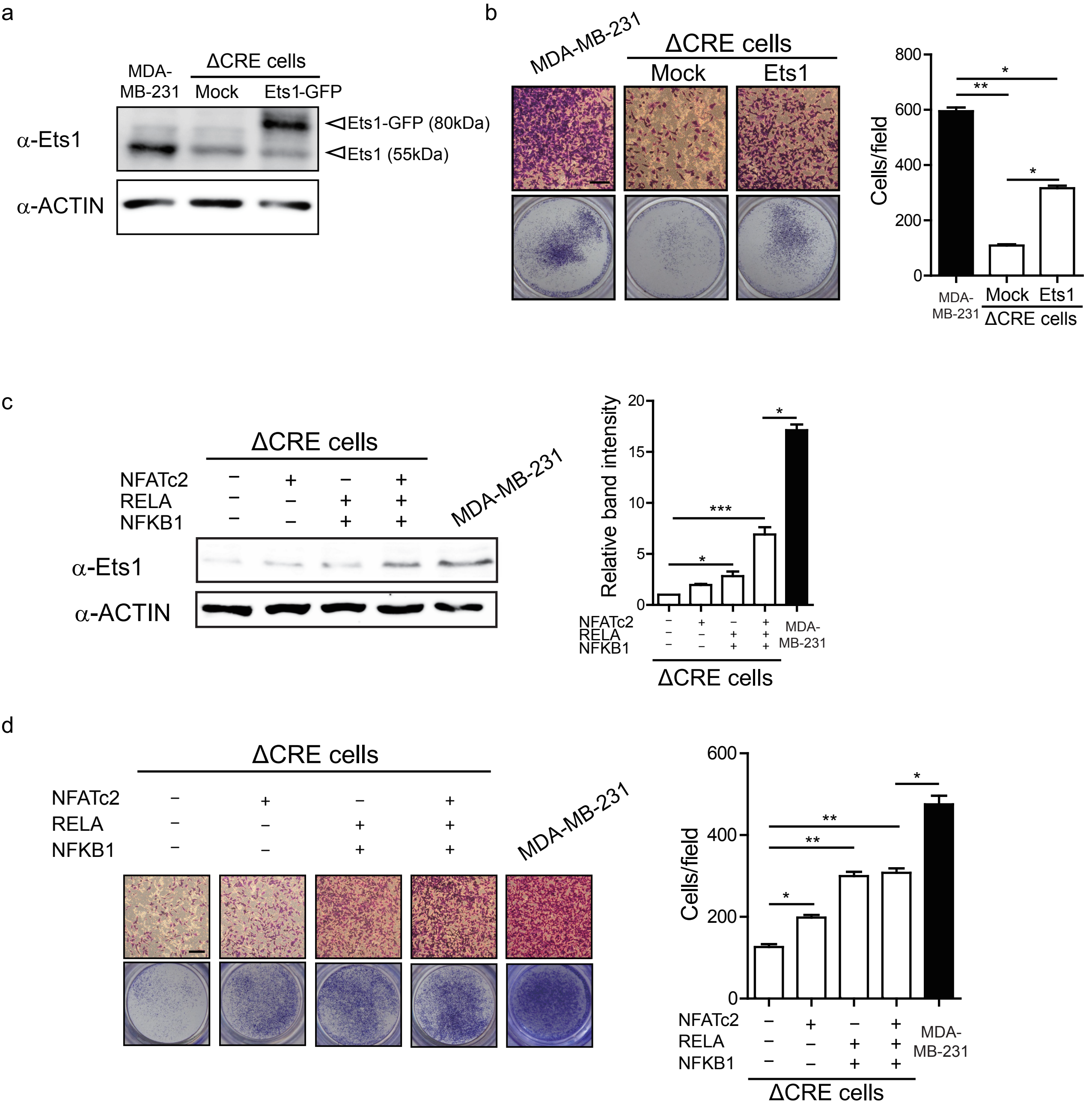

Supplementary Figure 7. Overexpression of NFATc2, NFKB1/RELA and Ets1 rescues invasion properties of ΔCRE cells. (a-b) Effect of Ets1 overexpression of on Ets1 level (a) and invasive properties in ΔCRE cells determined by Immuno-blot (a) and Invasion assay (b). (c-d) Effect of overexpression of NFATc2 and NFKB1/RELA on Ets1 expression and invasive properties in ΔCRE cells was determined by (c) Immuno-blot and (d) Invasion assay. Scale bar: 100μm. Relative band intensity of Immuno-blot was quantified by Image J software. Data shown are representative of more than three independent experiments with similar results. $^*P < 0.05$, $^{**}P < 0.01$ and $^{***}P < 0.001$

Table 1. Primer sequences used for qReal-Time PCR

Genes	Sequences (5' - 3')	
HPRT	F: CTGAAGAGCTATTGTAATGACCAG	R: TCTTTGGATTATACTGCCTGAC
Ets1	F: TCACTAAAGAACAGCAACGA	R: GGTTTCACATCCTCTTTCTG
NFATC2	F: TGCATCTAACCCCATCGAGTG	R: TGAGGATCATTTGCTGGC
NFKB1	F: TCTCTATGACCTGGATGACTC	R: GTTTCATGTCTCCTTGTGCT
RELA	F: ACA GGA GAA GGG ACG CCA T	R: GAA GCC CTA CAG ACG AGC TCA
ENG	F: GTCTCAAGACCAGGAAGTCCA	R: CGTGTGCGAGTAGATGTACC
MMP14	F: TGAGGATCTGAATGGAAATGAC	R: CATAAAGTTGCTGGATGCCC
DNMT3A	F: CTCTTTGATGGAATCGCTACAGG	R: CCACTCCTGGATATGCTTCTG
DNMT3B	F: CCC TCA CAC TCA GAT CAT CTT CT	R: GCT ACG ACG TGG GCT ACA G
APOBEC3C	F: AATCCACAGATCAGAAACCCGA	R: CAGTATGTCGTCGCAGAACC

Table 2. Primer sequences used for ChIP PCR

Genes	Sequences (5' - 3')	
Ets1	F: TCCACCGTTTCGGGAAAGTC	R: GTGAATGAATGATGTACACGCAC
ENG	F: CCTCAGCCACTAGAACAAACC	R: AGGTGCCACATACTGCTCTC
MMP14	F: CTGTGTTAATTGCAGGGAG	R: CTCCTCAGACAACTCCCAC
DNMT3A/3B	F: GAAAGTCCGTCTGATTCTCCA	R: CCTTCATCCACATGCCTCAC

Table 3. Primer sequences used for Pyrosequencing

Locus	Sequences (5' - 3')
Ets1(-375)-F	ATTATGTTGTTATTGGGAGGG
Ets1(-375)-R	5'biotin- ACCAATATACAAACTAAATACACAAACC
Ets1(-375)-S	GGGTGATTAAGTTTTTAAGA
Ets1(-534)-F	TGGGTGGGTTTTGAAAAATTGA
Ets1(-534)-R:	5'biotin- CTCCCAATAACAACATAATTTTCC
Ets1(-534)-S	ATTTTTAAAGTTTTTTGAGGAT
